# Comparison of Unhoused and Domiciled Patients Evaluated for Trauma in a Level II Trauma Center

**DOI:** 10.5811/westjem.43498

**Published:** 2026-01-09

**Authors:** Paul A. Silka, Miriam R. Elman, Margarida Bettencourt, Melissa Harte

**Affiliations:** * Providence Seaside Hospital, Department of Emergency Medicine, Seaside, Oregon; † Oregon Health & Science University-Portland State University, School of Public Health, Biostatistics & Design Program, Portland, Oregon; ‡ Unaffiliated; § Regional Medical Center of San Jose, San Jose, California

## Abstract

**Introduction:**

California has one of the highest rates of homelessness in the United States. Unhoused individuals often have complex medical and behavioral health disorders, frequently complicated by substance use disorders. They have a significant risk of sustaining traumatic injuries. This report compares unhoused and domiciled patients treated at our Northern California trauma center.

**Methods:**

In this retrospective analysis of trauma patients we used data extracted from our institution’s Trauma Quality Improvement Program Trauma Registry for January 1, 2019–April 22, 2022 and compared characteristics of unhoused and domiciled individuals. All unhoused patients in the registry were included in the analysis, as well as an equal number of domiciled patients who were randomly selected during the same time frame. We described and compared demographic and clinical characteristics.

**Results:**

Of 8,529 patients in the registry, 181 (2.1%) were unhoused, and we selected 181 domiciled patients to compare. Unhoused patients were more likely male (83% vs. 61%, P < .001) and younger (48.8 ± 12.3 vs. 55. 8 ± 23.7 years, P <.001). Both cohorts had similar Injury Severity Scores. However, unhoused patients had a higher rate of hospital admissions (76.8% vs. 61.9%, P <.001) and longer hospital stays than domiciled patients (4.0 [IQR 2.0–9.0] days vs. 3.0 [IQR 1.0–6.0] days, respectively; P = .02). A higher proportion of unhoused patients received alcohol-(85.6% vs. 74.6%, P = .01) and drug screening (56.4% vs. 30.4%, P < .001) than domiciled patients. Of those screened for urine drugs, unhoused patients had a higher positive rate (76.5% vs. 50.9%, P < .001). Unhoused patients were more frequently injured by assault (30.4% vs. 8.8%, P < .001) or pedestrian strike (21.5% vs. 3.3%, P < .001), whereas more domiciled patients were injured in falls (46.4% vs. 21.5%, P < .001) and motor vehicle accidents (29.8% vs. 8.3%, P < .001). Falls were most common in the oldest quartile for both groups. In both cohorts, a “sharp object” was the most common mechanism of assault injury (40.0% vs. 37.5%, respectively). Assault by firearm occurred in 14.5% of unhoused and 18.8% of domiciled patients. Overall mortality was 2.2%, with no significant difference between groups (1.7 vs. 2.8%, P = .70).

**Conclusion:**

Unhoused patients were predominantly younger males with a higher incidence of substance use disorder and greater likelihood of injuries from assault and pedestrian strikes. Falls and assault with a sharp object were common in both cohorts. Unhoused patients were admitted more often and stayed longer in the hospital. Understanding the complexities of these patients can guide local and regional prevention and treatment measures.

## INTRODUCTION

California is in crisis in terms of its homeless population^1^ with Santa Clara County alone hosting 10,028 people experiencing homelessness in 2022.^2^ Unhoused individuals have a high burden of medical comorbidities complicated by under-treated behavioral health disease, substance use disorders, and high rates of traumatic injury, often due to victimization.^3^–^5^ Previous studies have shown that unhoused individuals have a higher rate of hospital admission following traumatic injury than their housed counterparts with prolonged stays due to the complexity of their injuries and complications with discharge planning.^4^–^9^

Preventing and treating physical injury due to victimization among the unhoused is complex. Previous evidence demonstrates unique injury patterns in unhoused populations.^9^ Lack of shelter complicated by the comorbidities associated with homelessness make preventing assault challenging. These same comorbidities add to the complexity of treating injuries and require additional time by social services and case managers to assure that the recovery initiated in the hospital can continue after discharge.^8^ Unhoused survivors of violent injury are at a greater risk for violent re-injury and death than domiciled assault victims.^10^ Additionally, they are at increased risk of fatal drug or alcohol overdose following violent injury.^10^ It is because of these social and medical complexities that we sought to better understand our unhoused trauma patients.

In this paper we sought to describe the epidemiology, clinical characteristics, mechanisms of injury, and hospital use patterns of unhoused and domiciled patients treated at our Level II trauma center in a Northern California urban center. Understanding that there are regional variations to injury mechanisms and social services, this analysis is intended to inform clinicians and policymakers, facilitating targeted prevention initiatives as well as specific case management and social service strategies.^8^

## METHODS

We performed a retrospective analysis of patients evaluated in the emergency department (ED) at a Level II trauma center from January 1, 2019–April 22, 2022, using data from the Trauma Quality Improvement Program Trauma Registry. All patients on whom a trauma activation was initiated by emergency medical services (EMS) personnel based on the county’s trauma center criteria were entered into the registry by a trained data abstractor. Patients were also entered into the registry if a trauma consultation was obtained by the attending emergency physician for patients who met trauma center criteria but were transported by non-EMS conveyance or if EMS personnel did not initially perceive injuries as needing trauma activation. In addition to other patient and visit characteristics from the electronic health record, the registry contains patients’ housing status based on patient self-report to the medical center’s Patient Access Personnel. Unhoused status is defined according to the Substance Abuse and Mental Health Services Administration for patients who lack a permanent living arrangement. It is updated during the hospital course if a patient is unable to answer questions upon arrival to the ED and/or presented initially without identification. Unhoused status was specified by Patient Access Personnel who chose unhoused from the dropdown menu in the patient management information system.

**Population Health Research Capsule**

What do we already know about this issue?

*California faces a homelessness crisis. Unhoused patients are frequently victimized, often presenting to the emergency department with trauma*.

What was the research question?

*Does traumatic injury to unhoused patients differ from injuries to domiciled patients in our trauma center?*

What was the major finding of the study? *Unhoused patients were more commonly male, younger, admitted more often and stayed longer despite similar Injury Severity Scores*.

How does this improve population health?

*Understanding the complexities of trauma care for these populations can guide local and regional prevention and treatment measures*.

Data were extracted from the registry for patients presenting to the ED during the study period and de-identified. We included all unhoused patients meeting study criteria as well as an equal number of domiciled patients who were randomly selected using the Google random number generator. Patients and their clinical characteristics were abstracted from the registry by an unblinded author, deidentified, and stored in an Excel spreadsheet (Microsoft Corporation, Redmond, WA). No review of medical records was conducted. This study was granted exempt status and a waiver of informed consent by the Medical Centers Institutional Review Committee for the Protection of Human Subjects.

Extracted registry data included patient and clinical characteristics such as housing status, sex, age, and ED disposition. Patients were considered admitted if they were classified as an inpatient or placed in observation, whereas they were characterized as discharged if they were discharged from the ED, transferred, or left against medical advice. The unit that a patient was admitted to was at the discretion of the attending trauma surgeon. Additional clinical characteristics in the registry data were admission to the intensive care unit (ICU), length of stay (LOS) in the hospital, results of serum alcohol levels and drug screening, injury mechanism based on the *International Classification of Diseases, 10^th^ Rev*, (*ICD-10*), and Injury Severity Score (ISS).

We used descriptive statistics to assess the characteristics of our study sample. Categorical variables were summarized as frequency with percentage, continuous variables as mean with standard deviation or median with interquartile range as appropriate. We compared characteristics of domiciled and unhoused patients using *t*- or Wilcoxon rank-sum tests for normally distributed and skewed continuous variables, respectively, and χ^2^ or Fisher exact tests (where expected cell count was < 5) for categorical variables. Statistical significance was assessed at *P* < .05 and tests were two-sided. We performed analyses using R v4.3.1 (R Foundation for Statistical Computing, Vienna, Austria).

## RESULTS

Of the 8,529 patients in the four-year period reviewed in the registry database, we identified 181 (2.1%) unhoused patients for study inclusion. A corresponding 181 domiciled patients were randomly selected for comparison. Unhoused patients were more likely to be male (83% vs 61%, *P* < .001) and younger (48.8 ± 12.3 vs 55.8 ± 23.7 years of age, *P* < .001) than domiciled patients (Table 1).

Although no statistical difference was seen in ISS, unhoused patients had a higher proportion of hospital admissions (76.8% vs 61.9%, *P* < .001) than domiciled patients. There was no significant difference in ICU admissions between unhoused and domiciled patients (29.3% vs 24.3%, respectively, *P* =.29). Unhoused individuals had longer hospital stays than domiciled patients (4.0 [IQR 2.0–9.0] and 3.0 [IQR 1.0–6.0] days, respectively; *P* = .02). Unhoused patients were more likely to receive alcohol (85.6% vs 74.6%, *P* = .01) and drug screening (56.4% vs 30.4%, *P* < .001) than domiciled patients. Of those with screening results for drugs or alcohol, unhoused patients had a higher rate of positive urine drug screens (76.5% vs 50.9%, *P* < .001), but we found no difference in blood alcohol levels (46.5% vs 37.0% for unhoused and domiciled patients, respectively; *P* = .11). Blood alcohol and urine drug screens tended not to be performed or have missing data for domiciled patients.

Assessment of injury mechanisms indicates unhoused patients were more frequently injured due to assault (30.4% vs 8.8%, *P* < .001) or pedestrian strike (21.5% vs 3.3%, *P* < .001), whereas a greater percentage of domiciled patients were injured in falls (46.4% vs 21.5%, *P* < .001) and motor vehicle accidents (29.8% vs 8.3%, *P* < .001) (Table 2). Falls are most common in the oldest age quartile for both unhoused and domiciled patients. The greatest rate of unhoused patients injured by a pedestrian strike were seen in the youngest quartile, < 35 years of age (Table 2). In both unhoused and domiciled patients, a “sharp object” was the most common mechanism of injury during an assault, 40.0% and 37.5%, respectively. Assault by sharp object occurred most frequently in the quartile < 35 years of age (Table 2). Assault by firearm occurred in 14.5% of unhoused and 18.8% of our domiciled patients and was most frequently seen in the unhoused 35–49 years of age quartile where this mechanism of injury occurred in 7 of the 22 (31.8%) assaults (Table 2).

There was no difference in the mortality rate between the two groups (1.7% in unhoused vs 2.8% in domiciled, *P* = .70), which was 2.2% overall (Table 1).

## DISCUSSION

More than 2% of patients in this four-year analysis of trauma registry data were experiencing homelessness. This is higher than the 0.7% homeless rate reported in a similar analysis of the national Trauma Quality Improvement Program Trauma Registry data in the US and Canada.^9^ Our unhoused cohort was younger, predominantly male, more likely to be injured due to assault or being struck as a pedestrian, with a higher percentage of admissions to the hospital and a longer LOS than domiciled trauma patients randomly selected from the same registry during the study period. The unhoused patients were more likely to have laboratory alcohol and drug screening and had a higher proportion of positive urine drug screens. Previous studies have found comparable characteristics of unhoused patients seen in the emergency department (ED) setting, including those presenting with traumatic injuries.^4^,^8^,^9^ Despite these differences in demographic, utilization, and injury characteristics, the proportion of deaths in the two groups was similar.

An analysis of our results with regard to the mechanism of injury was performed with the intent of advancing our care and guiding injury prevention of our trauma patients, particularly in the unhoused. As with previously published work, we found falls to be a significant mechanism of injury in traumatic injuries for all trauma patients with the occurrence increasing with age and more common in the domiciled.^9^ Our unhoused cohort had a high rate of injury due to assault and pedestrian strikes (Table 2). Unhoused individuals injured as a pedestrian accounted for 21.8% of injuries in our unhoused cohort vs 3.3% of the domiciled. These injuries were most prevalent in our youngest unhoused age quartile, < 35 years of age, and is a phenomenon again seen on a national level, deserving of further investigation to develop preventive strategies.^3^,^4^,^9^

Both cohorts were frequently assaulted by sharp object(s), referred to here as “stabbing.” Stabbing accounted for 40% of assaults experienced by the unhoused and 37.5% of assaults in the domiciled group. Previously published literature has found stab wounds are experienced at a significantly greater rate in the unhoused population and consistently more often the mechanism of injury than injury from a firearm. 9,10 It has been hypothesized that there is a “hidden epidemic” of stabbing violence fueled by the sequelae of drug addiction.^11^ Accessibility of knives or other sharp instruments that can be weaponized makes it uniquely difficult to design preventative interventions. Meanwhile, violence intervention programs have focused on gun violence with less emphasis given to stabbing.^11^ Our analysis, showing a high rate of stabbing injury, suggests prevention efforts focused on this mechanism of injury could impact both unhoused and domiciled individuals in our region.

When assaulted, the rate of firearm injury in our population was 14.5% and 18.8% in the unhoused and domiciled, respectively. While injury including death due to firearms is prevalent among unhoused individuals, the data are nuanced. In the national Trauma Quality Improvement Program Trauma data analysis, firearm injuries were significantly more common in the unhoused vs domiciled, 5.2% and 4.1%, respectively.^9^ Additionally, non-fatal firearm injury has been seen more frequently in the unhoused population.^12^ Furthermore, death due to firearms has been found to be a leading cause of mortality in the unhoused.^3^,^13^ Other investigators found that overall homelessness was not associated with increased firearm homicide rates except in chronically unhoused individuals.^14^ Courtepatte found that stab wounds were more prevalent than gunshot wounds particularly in older, White, unhoused patients with pre-existing mental health and substance use disorders.^10^ It has been proposed that trauma centers continually review the populations they are serving to identify additional resources required to optimize care and adjust prevention efforts, as traumatic injuries are influenced by local factors.^8^,^9^

American College of Surgeons (ACS) guidelines have traditionally encouraged trauma centers to perform laboratory screening for the presence of alcohol when treating victims of physical trauma. Updated guidelines from the ACS now recommend adding a urine drug screen (UDS) as part of trauma care.^15^ We appropriately evaluated 80% of our trauma patients for alcohol use including equal numbers of housed and unhoused being tested. Only 57% of our trauma patients had UDS performed, with significantly more unhoused being tested and found to have positive screens at 43.3% (Table 1). Other investigators have found a similar phenomenon.^8^,^9^,^10^ The UDS has been considered of limited clinical value in the treatment of trauma patients given clinical ability to identify relevant toxidromes, while delays in obtaining test results add to the cost of care but minimally impact management.^16^ Criteria driving the decision to order a UDS based on age of the victim, mechanism, time of day, and day of the week has been demonstrated to reduce the number of negative studies ordered and the associated costs.^16^ The updated guidelines for urine drug screening are recommended for all trauma patients with the intent that individuals with positive screenings can undergo a “brief intervention” by trained coordinators and be referred for treatment.^15^

Our unhoused and domiciled cohorts had similar ISS; however, unhoused patients had a higher rate of inpatient admissions and LOS (Table 1). There was no difference in the rate of admission to the ICU between the two cohorts. Increased admission rates reflect the complexity of caring for these patients who in addition to their acute traumatic injuries often present with recent ingestion of drugs and alcohol confounding disposition decisions.^7^,^8^, Prolonged LOS is described for unhoused patients as there are numerous barriers impacting the decision to discharge, be it from the ED or an inpatient unit.^8^ Clinicians may perceive significant risk in discharging an unhoused trauma patient back to the street, a setting where the patient was initially injured.^9^ Discharging unhoused individuals from an inpatient unit after a trauma admission often requires extensive discharge planning and must consider post-discharge therapies and convalescence for care of acute injuries, in addition to undertreated co-morbidities including substance use disorder and behavioral health factors raising the concern that patients will be “lost to follow-up.”^7^,^8^

Results of our analysis demonstrate an unhoused population presenting with physical trauma that has a high rate of injury due to assault with concurrent substance use. This provides local experience that can guide injury treatment and targeted prevention efforts. The linkage between homelessness, substance use, behavioral health co-morbidities, and injury is prevalent throughout the literature.^4^,^5^,^7^,^9^,^17^ The prevalence of traumatic injuries among a growing number of unhoused nationally should stimulate further multidisciplinary, prospective investigations to determine effective injury prevention strategies. Work in this area has demonstrated how progressive case management in addition to intensive social services support are needed to secure post-acute care options such as respite care and referral to temporary housing, as well as concurrent behavioral health and addiction treatment.^10^ Further development of centralized homeless healthcare clinical services in a specialized setting, offering access to follow-up, primary care, addiction services, and behavioral and social services following trauma hospitalization has been demonstrated to reduce LOS, unplanned returns due to complications of injuries, and prevention of readmission from future injuries.^18^–^20^

## LIMITATIONS

Results from this single-center analysis may not be generalizable; however, demographic characteristics of our unhoused cohort are comparable to those described in other studies, suggesting our results may inform other centers.^4^,^5^,^7^,^8^,^9^ Additionally, we assume that accurate coding and complete data entry occurred for the registry used in the study. In particular, the number of unhoused patients in our analysis may be underestimated despite percentages described in similar studies using national trauma data, as patients who self-report housing status may be reluctant to disclose housing insecurity.^9^,^18^,^21^ The reality of individuals’ housing status is also likely much more complex and nuanced than we describe. For example, some unhoused persons may spend time split between shelters, hotels, and/or friends and family; conversely, authors have described the concept of “marginally housed.”^4^ Further, we do not have information about the onset or duration of homelessness.

Admission decisions, including ICU utilization and hospital discharge, were made at the discretion of the attending trauma surgeon. Some patients admitted to the ICU may have had medical indication for that level of care; however, that does not impact our chosen outcomes. Bias in this decision-making could impact some of the characteristics we report, including admission vs discharge from the ED, ICU utilization, and LOS. We were unable to obtain and compare hospital charges for our cohorts. We hypothesize that given the increased rate of admission and the longer LOS observed in the unhoused cohort we might have found our unhoused cohort care more expensive. Lastly, we chose to randomly select a subset of domiciled patients to compare with our unhoused cohort based on the resources available to conduct this study. Employing a different study design—eg, homeless and domiciled individuals matched by mechanism of injury, and/or substance use—would reduce the potential confounding effect of these important factors and allow for better elucidation of the association of homelessness with outcomes.

## CONCLUSION

In this analysis of the Trauma Quality Improvement Program Trauma Registry from our trauma center, unhoused individuals were predominantly younger males, having a higher rate of substance use and a greater likelihood of admission and longer length of stay despite similar Injury Severity Scale scores when compared to the domiciled cohort. The unhoused cohort had a greater likelihood of injuries resulting from assault and pedestrian strike than domiciled individuals. Falls were the predominant mechanism of injury in the domiciled cohort and were most common in the oldest age quartile of both cohorts. Assaults due to stabbing were prevalent in both cohorts, while injury due to firearms was less frequent but with a noteworthy spike in the unhoused 35–49 years of age quartile. Understanding the complexities of these patient populations can guide local and regional prevention and treatment measures. Prospective multidisciplinary investigations are needed to determine best practices for injury treatment, prevention, and discharge resources.

## Figures and Tables

**Table 1:** Demographic and clinical characteristics of 362 trauma patients by housing status in a 4-year analysis of trauma registry data.

Characteristic	Housing status		P-value

	Unhoused (n = 181)	Domiciled (n = 181)	
Age at ED visit, years, mean (SD)	48.8 (12.3)	55.8 (23.7)	< .001
Age group at ED visit			< .001
< 35 years	29 (16.0)	45 (24.9)	
35–49	53 (29.3)	33 (18.2)	
50–64	87 (48.1)	30 (16.6)	
≥65 years of age	12 (6.6)	73 (40.3)	
Female sex	32 (17.7)	73 (40.3)	< .001
ED visit year			.10
2019	38 (21.0)	55 (30.4)	
2020	50 (27.6)	45 (2*4.9)	
2021	67 (37.0)	50 (27.6)	
2022	26 (14.4)	31 (17.1)	
Any admission	139 (76.8)	112 (61.9)	< .001
Admission to ICU	53 (29.3)	44 (24.3)	.29
Hospital length of stay, days, median (IQR)	4.0 (2.0, 9.0)	3.0 (1.0, 6.0)	.02
Injury Severity Score			.30
Mild (≤ 8)	90 (52.0)	106 (62.4)	
Moderate (9–15)	64 (37.0)	48 (28.2)	
Severe (16–24)	9 (5.2)	9 (5.3)	
Extremely severe (≥ 25)	10 (5.8)	7 (4.1)	
Missing	8	11	
Blood alcohol screen			.01
Positive	72 (39.8)	50 (27.6)	
Negative	83 (45.9)	85 (47.0)	
Not performed/missing	26 (14.4)	46 (25.4)	
Drug screen			<.001
Positive	78 (43.3)	28 (15.5)	
Negative	24 (13.3)	27 (14.9)	
Not performed/missing	79 (43.6)	126 (69.6)	
Died	3 (1.7)	5 (2.8)	.70

* Data are n (%) unless otherwise noted.

ED, emergency department; ICU, intensive care unit.

**Table 2:** Mechanism of injury for 362 trauma patients by housing status and age group* in a 4-year analysis of trauma registry data.

Mechanism of Injury	Age < 35 years		Age 35–49 years		Age 50–64 years		Age ≥ 65 years		All Ages	

	Unhoused (n = 29)	Domiciled (n = 45)	Unhoused (n = 53)	Domiciled (n = 33)	Unhoused (n = 87)	Domiciled (n = 30)	Unhoused (n = 29)	Domiciled (n = 45)	Unhoused (n = 29)	Domiciled (n = 45)
Pedestrian injured in transport accident	10 (34.5)	1 (2.2)	9 (17.3)	2 (6.1)	19 (22.1)	2 (6.7)	1 (8.3)	1 (1.4)	39 (21.8)	6 (3.3)
Pedal cycle rider injured in transport accident	1 (3.4)	3 (6.7)	9 (17.3)	1 (3.0)	5 (5.8)	4 (13.3)	0 (0.0)	1 (1.4)	15 (8.4)	9 (5.0)
Motor vehicle accident	5 (17.2)	25 (55.6)	2 (3.8)	12 (36.4)	4 (4.6)	10 (33.3)	4 (33.3)	7 (9.6)	15 (8.3)	54 (29.8)
Motorcycle rider injured in transport accident	0 (0.0)	4 (8.9)	0 (0.0)	5 (15.2)	0 (0.0)	3 (10.0)	0 (0.0)	0 (0.0)	0 (0.0)	12 (6.6)
Car occupant injured in transport accident	4 (13.8)	19 (42.2)	2 (3.8)	7 (21.2)	3 (3.5)	4 (13.3)	3 (25.0)	5 (6.8)	12 (6.7)	35 (19.3)
Occupant of pick-up truck or van injured in transport accident	1 (3.4)	2 (4.4)	0 (0.0)	0 (0.0)	1 (1.2)	3 (10.0)	1 (8.3)	2 (2.7)	3 (1.7)	7 (3.9)
Other land-transport accidents	0 (0.0)	1 (2.2)	0 (0.0)	1 (3.0)	0 (0.0)	0 (0.0)	0 (0.0)	0 (0.0)	0 (0.0)	2 (1.1)
Slipping, tripping, stumbling, and falls	1 (3.4)	4 (8.9)	4 (7.7)	9 (27.3)	29 (33.7)	9 (30.0)	5 (41.7)	62 (84.9)	39 (21.8)	84 (46.4)
Exposure to inanimate mechanical forces	0 (0.0)	1 (2.2)	1 (1.9)	1 (3.0)	1 (1.2)	1 (3.3)	0 (0.0)	0 (0.0)	2 (1.1)	3 (1.7)
Exposure to animate mechanical forces	0 (0.0)	0 (0.0)	1 (1.9)	0 (0.0)	1 (1.2)	0 (0.0)	1 (8.3)	0 (0.0)	3 (1.7)	0 (0.0)
Accidental exposure to other specified factors	0 (0.0)	0 (0.0)	0 (0.0)	0 (0.0)	0 (0.0)	0 (0.0)	0 (0.0)	1 (1.4)	0 (0.0)	1 (0.6)
Intentional self-harm	0 (0.0)	1 (2.2)	0 (0.0)	0 (0.0)	2 (2.3)	0 (0.0)	0 (0.0)	0 (0.0)	2 (1.1)	1 (0.6)
Assault	11 (37.9)	6 (13.3)	22 (41.5)	6 (18.2)	21 (24.1)	4 (13.3)	1 (8.3)	0 (0.0)	55 (30.4)	16 (8.8)
Assault by rifle, shotgun, and larger firearm discharge	0 (0.0)	0 (0.0)	1 (1.9)	0 (0.0)	0 (0.0)	0 (0.0)	0 (0.0)	0 (0.0)	1 (0.6)	0 (0.0)
Assault by other and unspecified firearm and gun discharge	1 (3.4)	0 (0.0)	6 (11.5)	1 (3.0)	0 (0.0)	2 (6.7)	0 (0.0)	0 (0.0)	7 (3.9)	3 (1.7)
Assault by sharp object	6 (20.7)	3 (6.7)	6 (11.5)	2 (6.1)	10 (11.6)	1 (3.3)	0 (0.0)	0 (0.0)	22 (12.3)	6 (3.3)
Assault by blunt object	3 (10.3)	0 (0.0)	2 (3.8)	0 (0.0)	2 (2.3)	1 (3.3)	0 (0.0)	0 (0.0)	7 (3.9)	1 (0.6)
Assault by bodily force	1 (3.4)	2 (4.4)	6 (11.5)	3 (9.1)	7 (8.1)	0 (0.0)	1 (8.3)	0 (0.0)	15 (8.4)	5 (2.8)
Assault by other specified means	0 (0.0)	1 (2.2)	1 (1.9)	0 (0.0)	0 (0.0)	0 (0.0)	0 (0.0)	0 (0.0)	1 (0.6)	1 (0.6)
Assault by unspecified means	0 (0.0)	0 (0.0)	0 (0.0)	0 (0.0)	2 (2.3)	0 (0.0)	0 (0.0)	0 (0.0)	2 (1.1)	0 (0.0)
Event of undetermined intent	0 (0.0)	2 (4.4)	4 (7.7)	0 (0.0)	3 (3.5)	0 (0.0)	0 (0.0)	1 (1.4)	7 (3.9)	3 (1.7)
Legal Intervention	1 (3.4)	1 (2.2)	0 (0.0)	0 (0.0)	0 (0.0)	0 (0.0)	0 (0.0)	0 (0.0)	1 (0.6)	1 (0.6)
Toxic effects of substances, chiefly nonmedicinal as to source	0 (0.0)	0 (0.0)	0 (0.0)	1 (3.0)	0 (0.0)	0 (0.0)	0 (0.0)	0 (0.0)	0 (0.0)	1 (0.6)
Unreported/missing	0 (0.0)	0 (0.0)	1 (1.9)	0 (0.0)	2 (2.3)	0 (0.0)	0 (0.0)	0 (0.0)	3 (1.7)	0 (0.0)

* Data are n (%).
